# FGF-2 signaling dominance in TGF-β1/FGF-2–primed nasoseptal chondrocytes for cartilage tissue engineering

**DOI:** 10.1177/20417314261479435

**Published:** 2026-08-13

**Authors:** Zhiyao Ma, Kiarra Grimes, Aillette Mulet-Sierra, Melanie Kunze, Kezhou Wu, Adetola B. Adesida

**Affiliations:** 1 Department of Surgery, Division of Orthopaedic Surgery, 12357 University of Alberta, Edmonton, AB, Canada; 2 Sports Medicine Center, Department of Orthopaedics, The First Affiliated Hospital of Shantou University Medical College, Shantou, Guangdong, China; 3 Department of Biomedical Engineering, 3158 University of Alberta, Edmonton, AB, Canada; 4 Department of Biomedical Engineering, Shantou University, Shantou, Guangdong, China; 5 Sports Medicine Research Institute, Shantou University Medical College, Shantou, Guangdong, China; 6 Department of Surgery, Division of Otolaryngology, 12357 University of Alberta, Edmonton, AB, Canada; 7 Department of Surgery, Division of Surgical Research, 12357 University of Alberta, Edmonton, AB, Canada

**Keywords:** nasal chondrocytes, septal cartilage, cartilage tissue engineering, fibroblast growth factor 2 (FGF-2), transforming growth factor beta 1 (TGF-β1), microtissues, transcriptomics

## Abstract

Nasoseptal chondrocytes (NCs) are a viable cell source for engineering autologous hyaline cartilage grafts for nasal reconstruction or repairing articular cartilage lesions. Fibroblast growth factor (FGF)-2 and transforming growth factor (TGF)-β1 are commonly used to optimize cell yield and chondrogenic capacity during *in vitro* culture. However, the comprehensive molecular effects of these factors have yet to be elucidated. Leveraging RNA sequencing, this study characterizes the individual and interactive effects of FGF-2 and TGF-β1 on the NC monolayer transcriptome and subsequent 3D cartilage microtissue qualities, revealing that dual-primed NC transcriptomes converge on an FGF-2-like state. Altogether, our analysis of growth factor interactions at the molecular level provides novel, foundational insights into the regulation of intracellular signaling pathways essential to advancing tissue engineering strategies.

## Introduction

To address the limited intrinsic healing capacity of hyaline cartilage, a valuable and versatile source for tissue engineering may be right under our noses. Nasal septal cartilage is an anterior quadrilateral plate of flexible hyaline cartilage, providing critical structural support to the upper airway and dividing the nasal cavity into two nostrils.^1^ For surgical reconstruction of the nasal alar cartilage after excision of non-melanoma skin cancer and repair of articular cartilage lesions in the knee joint, autologous nasal septal cartilage has emerged as a potentially optimal cell source.^2–5^ The safety and feasibility of nasoseptal chondrocytes (NCs) for generating cartilage grafts in both applications have been established in clinical trials by researchers at the University of Basel, Switzerland.^6–8^ While these studies have achieved successful functional outcomes and patient satisfaction, significant inter-patient variability and low cell yield from donor tissue have been identified as primary threats to the predictability and scalability of these procedures.^2,6^ Although recent advances have aimed to enhance quality prediction of NC-generated grafts via biomarker identification,^9,10^ the challenge of reliably generating a sufficient number of cells capable of producing viable neocartilage remains unresolved. As such, NC tissue engineering methods must be optimized to consistently and efficiently generate high-quality grafts.

The combination of two growth factors: fibroblast growth factor (FGF)-2 and transforming growth factor (TGF)-β1, has been predominantly used to enhance the quality and quantity of engineered cartilage tissues.^11–15^ Both factors are important for homeostasis of the extracellular cartilage matrix and intracellular regulation of growth, proliferation, and chondrocyte-specific expression.^11,16,17^ In the absence of native physiological cues *in vitro*, exogenous growth factors are introduced to regulate cellular signalling.^18–20^ FGF-2 is primarily used to stimulate proliferation during the cell expansion phase to overcome the relatively low intrinsic proliferative rate of NCs, although this often occurs at the cost of dedifferentiation into a more fibroblast-like phenotype.^13,21,22^ TGF-β1 is regarded for its ability to maintain a chondrogenic phenotype *in vitro*,^23,24^; thus, the two factors in combination contribute to the production of hyaline cartilage-like tissue constructs of ample volume for clinical use. Compared with either factor alone, their combined use has been reported to enhance cartilage-forming capacity-related characteristics, such as construct size, glycosaminoglycan (GAG) accumulation, and collagen II deposition.^13,14,25^ While prior work has largely been limited to tissue-level analyses of these growth factors,^12–14^ their combined and individual effects on global cellular signalling have not been comprehensively studied. Here, we leverage unbiased RNA sequencing to address this gap and delineate the transcriptomic profile of each priming condition, including the extent to which these are maintained or reprogrammed during the transition from 2D monolayer to 3D tissue composition. While clinical implementation of NCs for tissue engineering holds great potential in regenerative medicine, it requires an in-depth understanding of how growth factors and culture conditions influence cartilage tissue formation, as we aim to provide in this study.

## Methods

### Ethics and donor information

Human nasal septal cartilage was obtained from discarded septorhinoplasty specimens of six donors under University of Alberta Health Research Ethics Board-Biomedical Panel approval (Study ID: Pro18778). Donors included three males aged 17, 36, and 42 years, and three females aged 19, 34, and 57 years. All procedures were performed in compliance with University of Alberta guidelines and applicable regulations.

### Cartilage collection and storage

Nasal septal cartilage was harvested from each donor and transported to the laboratory in sterile buffered saline. When immediate processing was not feasible, the tissue was transferred to complete Dulbecco’s modified Eagle medium (DMEM) containing 10% fetal bovine serum (FBS) (F1051, Sigma-Aldrich), 1% w/v penicillin-streptomycin-glutamine (PSG) (10378, Gibco), and 1% w/v HEPES (H4034, Sigma-Aldrich), and maintained under normoxic conditions (21% O_2_, 5% CO_2_, 37 °C) for up to 1 week. For same-day isolation, cartilage was briefly washed in Dulbecco’s phosphate-buffered saline (DPBS) supplemented with 1% PSG to remove surface debris prior to enzymatic digestion.

### Nasoseptal chondrocyte isolation

Under aseptic conditions, cartilage was dissected into approximately 1-2 mm^3^ fragments and weighed in sterile 50 mL tubes, with no more than 2 g of cartilage per tube. A freshly prepared collagenase type II (LS004176, Worthington) working solution (∼450 U/mL) was added at approximately 10 mL per gram of tissue, and samples were digested for 16-22 h at 37 °C with gentle shaking (250 rpm) to dissociate the cartilage matrix. Collagenase digestion was neutralized with an equal volume of complete DMEM (D6429, Sigma-Aldrich) supplemented with 10% v/v FBS, 1% w/v PSG, and 1% w/v HEPES. The digest was filtered through a 100 μm cell strainer to remove residual debris, and the filtrate was centrifuged at 1500 rpm for 10 min at room temperature. The resulting cell pellet was resuspended in fresh complete DMEM, briefly washed in DPBS, and used for expansion.

### Cell expansion

Freshly isolated nasoseptal chondrocytes (NCs) were seeded into T75 or T175 flasks and cultured under normoxic conditions (21% O_2_, 5% CO_2_, 37 °C). No medium change was performed during the first 48 hours to support cell attachment. After this initial recovery period, flasks were gently rinsed with DPBS to remove non-adherent cells and debris. Adherent cells were detached with 0.05% w/v trypsin-EDTA (25051CI, Corning) for approximately 5 min at 37 °C and reseeded at 10,000 cells/cm^2^ in complete DMEM. Cultures were monitored for morphological changes and passaged again when 80-90% confluence was reached. Three growth factor groups were evaluated: DMEM supplemented with 1 ng/mL TGF-β1 (T1), 5 ng/mL FGF-2 (F2), or 1 ng/mL TGF-β1 plus 5 ng/mL FGF-2 (T1F2). Passage 2 (P2) cells were reseeded under their respective growth factor conditions and harvested at confluence for chondrogenic differentiation or suspended in (ThermoFisher, USA) at −80°C for molecular analyses. Cumulative population doublings throughout both passages were calculated using the following equation:

(1) $$\sum \log_{2}\left(\frac{Final\;cell\;count}{Initial\;cell\;count}\right)$$

### Chondrogenic differentiation

At the end of P2, cells from each growth factor condition were collected, and microtissue pellets were formed at 0.5 × 10^6^ cells per pellet by centrifugation at 1500 rpm for 10 minutes. Pellets were cultured in serum-free chondrogenic medium composed of high-glucose DMEM, 1× ITS+ (354352, Corning, Canada), 1 ng/mL TGF-β3 (ProSpec, Israel),^26–28^ 100 nM dexamethasone, 50 μg/mL ascorbic acid 2-phosphate, 125 μg/mL human serum albumin, 40 μg/mL L-proline, 1% PSG, and 1% HEPES. Three-dimensional cultures were maintained under normoxic conditions (21% O_2_, 5% CO_2_, 37 °C) for 3 weeks, with medium changes twice weekly. After 3 weeks of differentiation, microtissue pellets were harvested for morphological, mechanical, histological, immunofluorescence, and biochemical analyses.

### Histological and immunofluorescence analysis

Constructs were fixed in 10% (v/v) neutral-buffered formalin, dehydrated through a graded ethanol series (70%-100%), embedded in paraffin, and sectioned at 5 μm. Sections were deparaffinized in XS-3 xylene substitute (StatLab, USA), rehydrated through descending ethanol concentrations (100%, 70%, and 50%), and rinsed in distilled water. Safranin O/Fast Green staining was used to evaluate sulfated glycosaminoglycan (sGAG) deposition and extracellular matrix organization. Images were acquired with a Nikon Eclipse TI-S microscope and quantified via a systematic process employing a self-developed algorithm. Quantitative measurements included background subtraction to minimize nonspecific staining artifacts, calculation of the total pixel intensity for Saf-O, and measurement of the section area. The quantification result was expressed as the total Saf-O intensity over the pellet section area.

For immunofluorescence, sections were incubated overnight at 4 °C with primary antibodies diluted 1:200, namely rabbit anti-human type I collagen (CL50111AP, Cedarlane, Canada) and mouse anti-human type II collagen (II6B3, Developmental Studies Hybridoma Bank (DSHB), USA). On the second day, sections were incubated for 45 minutes with species-specific secondary antibodies, goat anti-rabbit IgG Alexa Fluor 594 (ab150080, Abcam) and goat anti-mouse IgG Alexa Fluor 488 (ab150117, Abcam) at a 1:200 dilution. Nuclei were counterstained with DAPI for 10 minutes, and fluorescence images were captured using a Nikon Eclipse TI-S microscope.

### Mechanical testing

To calculate the equilibrium modulus of cultured microtissue pellets, a stepwise series of stress-relaxation tests at 10%, 20%, and 30% tissue strain was conducted using a MicroTester G2 (CellScale Biomaterials Testing, Canada). The microtissue pellets were placed on a stable platform and compressed by a parallel upper platen. A sequence of 3 levels of 10% strain, ramping at 2% strain per second, was applied, each followed by a 300-second relaxation period at constant strain, during which all pellets reached equilibrium. The pellet diameter and upper platen height were recorded at the initial point of contact, and the device recorded changes in height and reaction forces over time throughout the testing sequence. The cross-sectional area of the pellet was calculated using the measured diameter, from which the stress with respect to the normalized recorded force was computed. The slope of the stress-strain curve resulting from linear regression analysis of the best-fit region was used to define the equilibrium modulus for each pellet.

### Biochemical assays

To assess extracellular matrix (ECM) composition, cultured pellets were digested overnight at 56 °C in proteinase K solution (P2308, Sigma-Aldrich). Total GAG content was quantified using the 1,9-dimethylmethylene blue (DMMB) (341008, Sigma-Aldrich, Canada) assay using chondroitin sulfate (C8529, Sigma-Aldrich, Canada) as the standard. DNA levels were determined using a CyQuant cell proliferation assay kit (C7026, ThermoFisher Scientific), employing various dilutions of bacteriophage λ DNA as the standard.

### RT-qPCR

For gene expression analysis, cell monolayers and microtissue pellets (n = 2 per donor per condition) were stored in TRIzol at -80 °C until RNA extraction. Total RNA was extracted and purified using the PuroSPIN Total RNA Purification Kit (Luna Nanotech, Canada) according to the manufacturer’s instructions. RNA concentration and purity were measured using a NanoDrop One C spectrophotometer (Thermo Fisher Scientific, Canada). Approximately 100 ng of total RNA was reverse-transcribed into cDNA using GoScript Reverse Transcriptase kit (Promega, Canada). Gene expression was quantified by RT-qPCR using gene-specific primers (Supplementary Table 1) and Takyon No-ROX SYBR MasterMix dTTP Blue (Eurogentec, Anaspec, USA). Expression levels were normalized to the average of Ribosomal Protein L13a (*RPL13A*), Tyrosine 3-monooxygenase/tryptophan 5-monooxygenase activation protein zeta protein (*YWHAZ*), and β-2-microglobulin (*B2M*) housekeeping genes, and expression level were calculated using the 2^−ΔCT^ method. For bulk RNA sequencing, the extracted total RNA samples were shipped to the University of British Columbia Biomedical Research Centre (UBC-BRC) for next-generation sequencing on the Illumina NextSeq 2000 platform.

### Bioinformatics analysis

Raw FASTQ files were analyzed in Partek Flow software (version 10.0.21.0302; Partek Inc., St. Louis, MO, USA) using an established pipeline. Bases with Phred quality scores <20 were trimmed from read ends, and the remaining high-quality reads were aligned to the human reference genome (hg38) using STAR (v2.7.8a). Reads were quantified against the Ensembl_release111_v2 annotation. Features with a maximum expression value ≤10 were filtered out before normalization. Expression values were offset by 1, normalized using the trimmed mean of M-values (TMM) method, and log2-transformed for downstream analysis. Differential expression analysis was performed in Partek Flow using the Limma-trend method. Genes were considered differentially expressed at an absolute fold change >2 and a false discovery rate (FDR) <0.05. Functional enrichment analyses, including Kyoto Encyclopedia of Genes and Genomes (KEGG) and Gene Ontology (GO) analyses, were also performed in Partek Flow. The KEGG network model was generated from the top enriched KEGG pathways. Significantly regulated genes associated with these pathways were identified from the KEGG tab in Supplementary Table 2 and grouped according to their known functional roles. These pathway-associated genes were then organized into KEGG modules and assembled based on their predicted subcellular or cellular-level site of action to illustrate the functional relationships among the enriched pathways.

### Statistical analysis

All statistical analyses other than the RNA-seq bioinformatics workflow were performed using GraphPad Prism (version 10.1.2). Outliers were identified using the ROUT method (Q = 1%). Normality was assessed using the Shapiro-Wilk test. For normally distributed data, one-way ANOVA with Geisser-Greenhouse correction and Tukey’s multiple-comparison test was used. For non-normally distributed data, the Friedman test with Dunn’s multiple-comparison test was applied. Correlations between monolayer and microtissue pellet gene expression measured by PCR were assessed using simple linear regression. Statistical significance was defined as *p < 0.05 and **p < 0.01, ***p < 0.001.

## Results

### Dual priming maximizes monolayer expansion, whereas FGF-2 priming generates the largest 3D pellets

**Figure 1.**
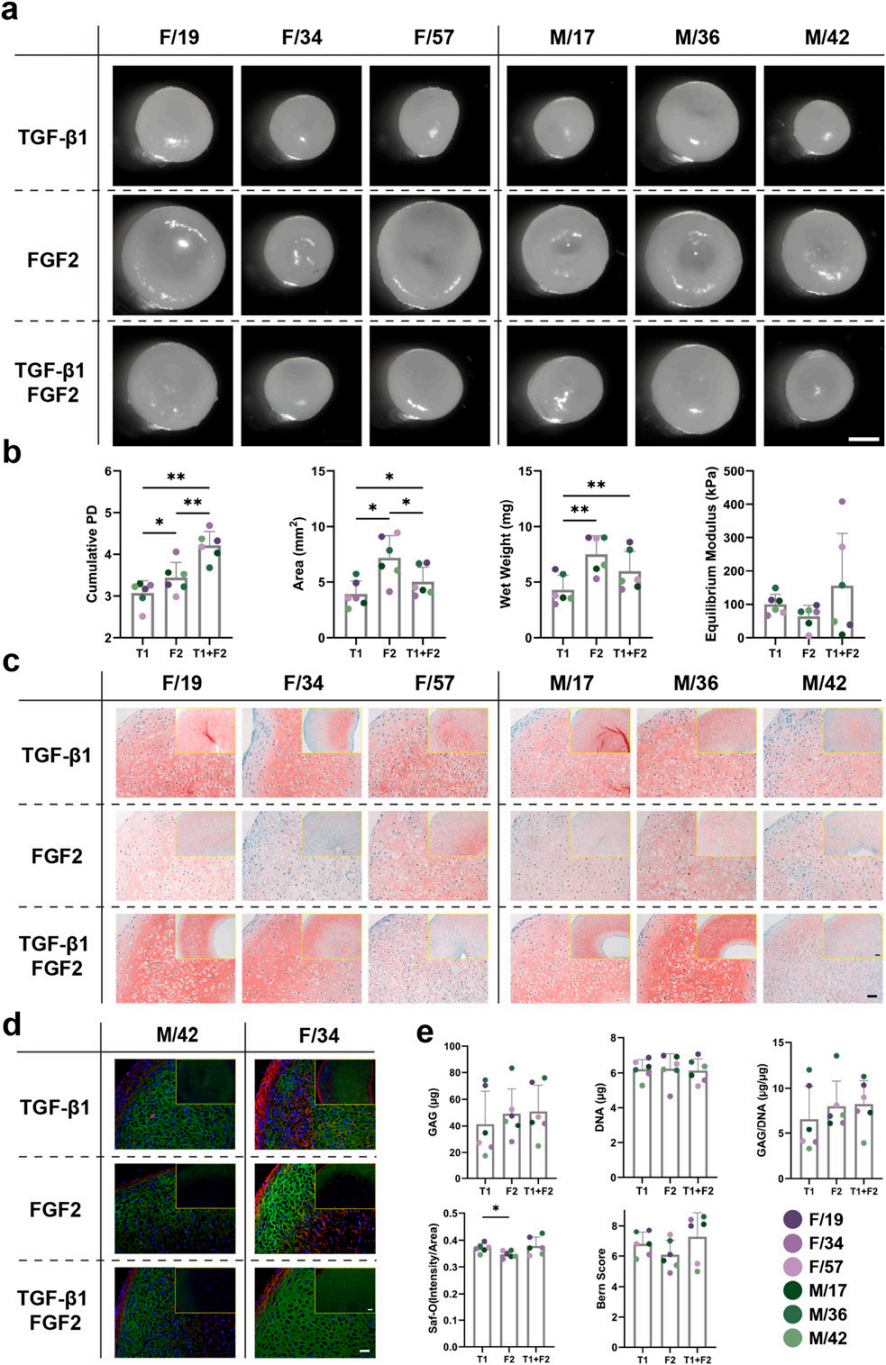
Biochemical and histological analyses of microtissue pellets formed from nasoseptal chondrocytes expanded with TGF-β1 and/or FGF-2. (a) Representative gross morphology images of pellets from all six donors (F/19, F/34, F/57, M/17, M/36, and M/42). Scale bar: 1mm. (b) Cumulative population doublings during monolayer expansion, pellet cross-sectional area, wet weight, and equilibrium modulus after 21 days of pellet culture. Scale bar: 100μm, insert: 50μm. (c) Representative Safranin-O/Fast-Green staining of donor-matched pellets. (d) Representative immunofluorescence images from donors (M/42 and F/34) showing collagen II (green), collagen I (red), and nuclei (blue). Scale bar: 100μm, insert: 50μm. (e) Biochemical and histological quantification of GAG, DNA, GAG/DNA, Safranin-O intensity normalized to pellet area, and Bern score. Bars indicate group means with individual donors overlaid. *p < 0.05, **p < 0.01.

Human NCs expanded under T1, F2, or dual T1+F2 priming showed condition-dependent differences in 2D proliferation. By P2, cumulative population doubling was highest in the dual-primed group (4.4 ± 0.2), followed by F2 (3.4 ± 0.2) and T1 (3.0 ± 0.1); dual priming exceeded both single-factor conditions, and F2 also exceeded T1 (Figure 1(b)). After 3 weeks of pellet culture, all groups formed rounded pellets with broadly similar gross morphology apart from size (Figure 1(a)). F2-primed pellets displayed the largest cross-sectional area with loss of volume in the pellet center, T1-primed pellets were the smallest, and T1+F2 pellets were intermediate, with all pairwise differences significant (Figure 1(b)). Wet weight followed a similar overall pattern; however, only T1 versus F2 and T1 versus T1+F2 were significantly different, whereas F2 and T1+F2 were not (Figure 1(b)). Mechanical properties of the tissue constructs, as reflected by the equilibrium moduli, did not differ significantly between groups but showed substantial donor-to-donor variability in the T1+F2 condition (Figure 1(b)).

### Dual-primed pellets show qualitatively denser cartilage-like matrix, while quantitative measurements remain comparable across groups

Safranin-O/Fast-Green staining revealed donor-dependent differences in matrix deposition (Figure 1(c)). F2-primed pellets generally showed weaker or less uniform Safranin (Saf)-O staining, whereas T1 and especially T1+F2 pellets more often displayed stronger cartilage-like matrix staining, although this trend was not uniform across all donors. Quantification of Saf-O staining showed T1 Saf-O intensity is significantly higher than F2 normalized to pellet area (Figure 1(e)). Representative immunofluorescence images from F/34 and M/42 showed collagen II distributed throughout the pellet interior, while collagen I was localized mainly to superficial/peripheral regions; T1+F2 sections appeared qualitatively denser in collagen II staining (Figure 1(d)). Despite these qualitative differences, total GAG content, DNA content, GAG/DNA ratio, and Bern score did not differ significantly among priming conditions (Figure 1(e)).

### Priming-dependent transcriptional differences are evident in P2 monolayers but largely converge after pellet formation

**Figure 2.**
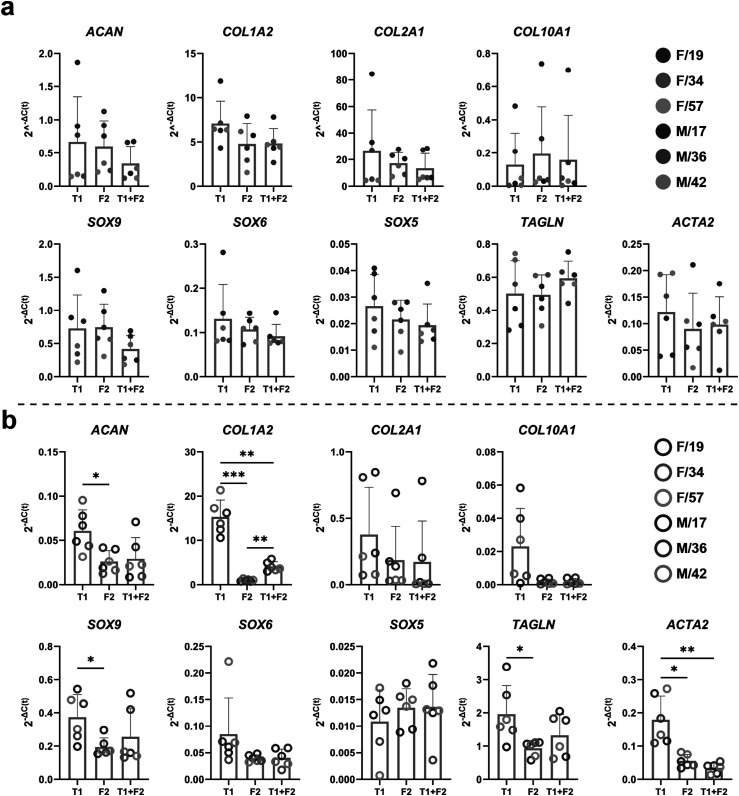
RT-qPCR analysis of donor-matched nasoseptal chondrocytes expanded with TGF-β1 and/or FGF-2. Expression is shown as 2^-ΔCt^. (a) Gene expression in pellets after 21 days of chondrogenic culture for *ACAN, COL1A2, COL2A1, COL10A1, SOX9, SOX6, SOX5, TAGLN*, and *ACTA2*. (b) Gene expression in P2 monolayer cells harvested at the end of expansion, before pellet induction, for the same gene panel. Bars indicate group means with individual donors overlaid. *p < 0.05, **p < 0.01, ***p < 0.001.

RT-qPCR analysis showed that, after 21 days of pellet culture, expression of chondrogenic transcription factors (*SOX9*, *SOX5*, and *SOX6*), matrix-associated genes (*ACAN*, *COL2A1*, and *COL1A2*), the hypertrophic marker *COL10A1*, and cytoskeletal genes (*TAGLN* and *ACTA2*) did not differ significantly among pellets generated from T1-, F2-, or T1+F2-primed NCs (Figure 2(a)). Donor-to-donor variability remained evident, but the transcriptional differences associated with priming during monolayer expansion were largely attenuated after 3D culture.

In contrast, clear differences were present in P2 monolayer cells before pellet formation (Figure 2(b)). T1-primed cells expressed significantly higher levels of *ACAN* and *SOX9* than F2-primed cells. *COL1A2* expression was highest in T1, lowest in F2, and intermediate in T1+F2, with significant differences between all three groups. *TAGLN* and *ACTA2* expression were also higher in T1 than in F2, and *ACTA2* was higher in T1 than in T1+F2. *COL10A1* transcripts were detected predominantly in T1 monolayers, although this difference was not significant. No significant differences were observed for *COL2A1*, *SOX6*, or *SOX5*. Collectively, these data indicate that priming-dependent transcriptional differences were most apparent at the end of monolayer expansion and largely converged after pellet formation.

### Monolayer-to-pellet transcript relationships are gene- and priming-dependent

**Figure 3.**
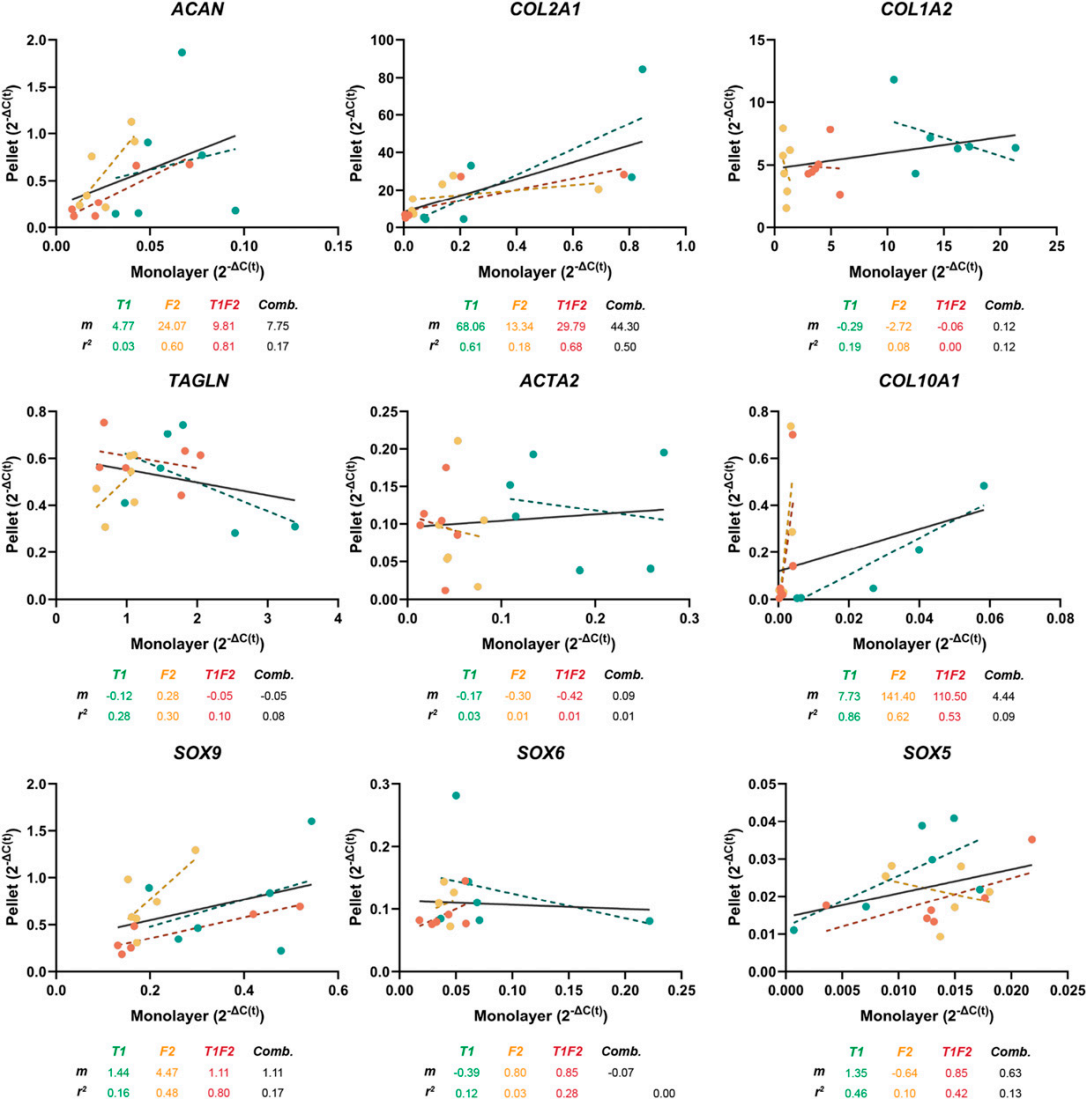
Monolayer-to-pellet gene expression relationships. Linear regressions comparing donor-matched monolayer and pellet gene expression (2^^-ΔCt^) for *ACAN, COL2A1, COL1A2, TAGLN, ACTA2, COL10A1, SOX9, SOX6*, and *SOX5*. Monolayer values were measured in P2 cells at the end of expansion; pellet values were measured after 21 days of chondrogenic culture. Colored dashed lines indicate within-group regressions for T1**,** F2**,** and T1+F2**;** the solid black line indicates the pooled regression across all groups. For each gene, the slope (m) and coefficient of determination (r^2^) are shown for individual treatment groups and pooled data.

To determine whether transcriptional differences present at the end of monolayer expansion were retained after pellet culture, donor-matched monolayer and microtissue pellet 2^-ΔCt^ values were compared by linear regression within each priming group (Figure 3). These analyses revealed gene- and treatment-dependent monolayer-to-pellet associations rather than a universal relationship across all transcripts.

Positive monolayer-to-pellet associations were most evident for *ACAN* in the F2 and T1+F2 groups (r^2^ = 0.60 and 0.81, respectively), but not in T1 (r^2^ = 0.03). *SOX9* showed a similar pattern, with a weak association in T1 (r^2^ = 0.16), a moderate association in F2 (r^2^ = 0.48), and the strongest association in T1+F2 (r^2^ = 0.80). In contrast, *COL2A1* expression was more correlated between monolayer and pellet in T1 and T1+F2 (r^2^ = 0.61 and 0.68, respectively) than in F2 (r^2^ = 0.18). *SOX5* also showed moderate positive relationships in T1 and T1+F2 (r^2^ = 0.46 and 0.42, respectively), with little evidence of association in F2 (r^2^ = 0.10). *COL10A1* displayed positive within-group relationships across all priming conditions, with the highest r^2^ in T1 (0.86), followed by F2 (0.62) and T1+F2 (0.53). By contrast, *COL1A2*, *TAGLN*, *ACTA2*, and *SOX6* showed weak or inconsistent monolayer-to-pellet associations across conditions. When all priming groups were pooled, most relationships weakened, although a moderate combined association remained for *COL2A1* (r^2^ = 0.50). Together, these findings suggest that whether monolayer gene expression tracks subsequent pellet expression depends on both the transcript examined and the expansion regimen used.

### FGF-2 biases the dual-primed monolayer transcriptome toward an F2-like state

**Figure 4.**
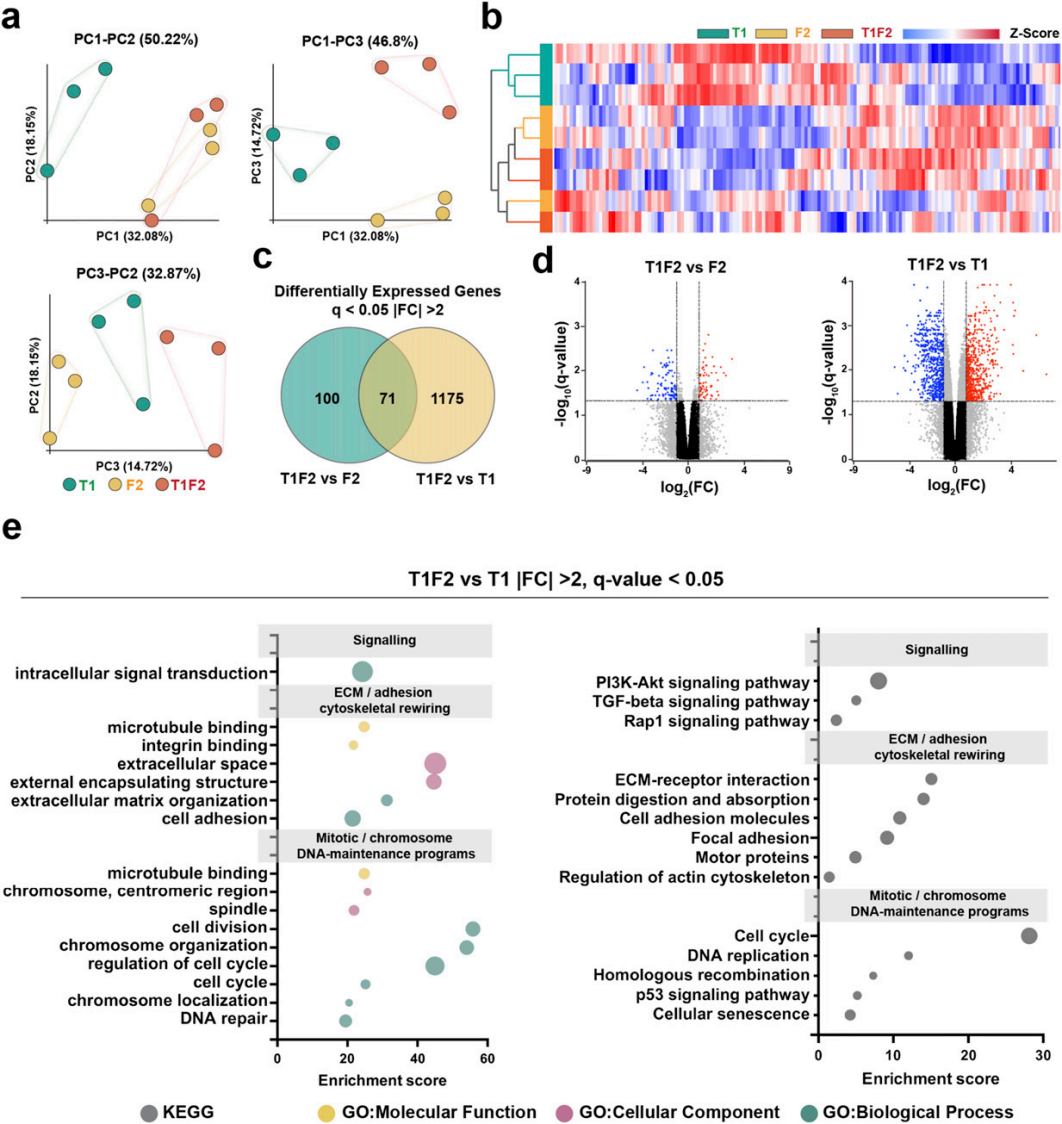
Transcriptomic characterization and functional enrichment analysis of growth factor priming by bulk RNA sequencing. Bulk RNA-seq was performed on donor-matched P2 monolayer nasoseptal chondrocytes from the sequenced donor subset (F/19, F/34, and M/42) expanded with TGF-β1 (T1) and/or FGF-2 (F2). (a) Principal component analysis showing pairwise projections of PC1-PC2, PC1-PC3, and PC3-PC2; the first three principal components together explain 64.95% of total variance. (b) Hierarchical clustering heatmap of transcriptomic profiles across the nine libraries. (c) Venn diagram showing overlap between differentially expressed genes (DEGs) in T1F2 vs F2 and T1F2 vs T1 comparisons using q < 0.05 and |FC| > 2. (d) Volcano plots for the same pairwise comparisons; positive log2(FC) values indicate genes increased in T1F2 relative to the comparator, and negative values indicate genes decreased in T1F2. (e) Selected non-redundant GO terms and enriched KEGG pathways for the T1F2 vs T1 comparison, grouped into three functional modules: mitotic/chromosome/DNA-maintenance programs, ECM/adhesion/cytoskeletal rewiring, and signaling. KEGG terms are shown in grey; GO terms are color-coded by ontology.

Bulk RNA-seq was performed on donor-matched P2 monolayer NCs from three sequenced donors (F/19, F/34, and M/42) expanded under T1, F2, or T1F2 conditions. After filtering and normalization, 19,899 genes were retained for analysis. Principal component analysis separated T1 from the two F2-containing groups, with T1F2 localizing closer to F2 than to T1, and hierarchical clustering showed the same overall relationship (Figure 4(a) and (b)). Consistently, differential expression analysis identified 1246 DEGs in T1F2 versus T1 but only 171 DEGs in T1F2 versus F2 (q < 0.05, |FC| > 2), with 71 genes shared between the two comparisons (Figure 4(c) and (d)). The T1F2 versus F2 functional enrichment analysis is also provided in Supplementary Figure 1 to emphasize the comparatively low enrichment scores of the identified modules, which correspond to a very limited number of DEGs under these terms. Together, these data indicate that the dual-primed monolayer transcriptome more closely resembles F2 than T1.

### FGF-2 reorganizes proliferative, matrix-engagement, and signaling programs

Because the larger transcriptional shift was observed in the T1F2 versus T1 comparison, downstream enrichment analysis focused on this contrast. While the T1F2 vs F2 comparison demonstrates much greater molecular congruence, subtle but coordinated shifts across functional categories were noted (Supplementary Figure 1). GO and KEGG terms converged on three major biological modules (Figure 4(e), Table 1, Supplementary Table 2). The strongest module comprised mitotic, chromosomal, and DNA-maintenance programs, including cell-cycle, DNA-replication, DNA-repair, spindle, and chromosome-organization terms. These categories were overwhelmingly dominated by decreased expression in T1F2 relative to T1, including checkpoint and mitotic regulators such as *CDC20* (FC -8.91), *PLK1* (-7.30), *AURKB* (-6.14), *CCNB1* (-5.49), *CDK1* (-3.58), together with replication-associated genes *MCM5* (-3.21), *POLE2* (-3.34), and kinesin/spindle-associated genes *KIF18B* (-10.55), indicating broad attenuation of replication, checkpoint control, and chromosome maintenance rather than a narrow change in proliferation alone.

**Table 1.** Functional modules by GO and KEGG, and signature genes with fold change.

Functional module	Representative GO/KEGG terms	Signature genes, FC (T1F2 vs T1)	
**Mitotic/chromosome/DNA-maintenance**	Cell cycle,	*MCM4*	-2.93
	DNA replication,	*MCM5*	-3.21
	DNA repair,	*POLE2*	-3.34
	**chromosome organization, spindle,**	*CDK1*	-3.58
	motor proteins	*CENPE*	-4.27
		*CCNB2*	-4.88
		*CCNB1*	-5.49
		*AURKB*	-6.14
		*PLK1*	-7.3
		*CDC20*	-8.91
		*KIF2C*	-7.77
		*KIF18B*	-10.55
**Structural ECM/adhesion**	ECM-receptor interaction,	*ITGA8*	6.86
	focal adhesion,	*ITGB8*	6.13
	cell adhesion,	*ITGA10*	2.58
	extracellular matrix organization,	*ITGA5*	-2.02
	integrin binding	*FN1*	-2.87
		*COL1A2*	-2.98
		*TNC*	-3.06
		*THBS1*	-3.18
		*COL1A1*	-4.29
		*ITGA11*	-4.89
		*COL4A1*	-5.5
		*COMP*	-9.85
**Matrix turnover/extracellular factors**	Extracellular matrix organization,	*C3*	41.48
	extracellular space,	*CHI3L1*	36.46
	external encapsulating structure	*A2M*	31.68
		*C2*	14.72
		*MMP10*	9.17
		*MMP13*	8.9
		*SERPING1*	5.14
		*CTSS*	4.27
		*FBLN1*	4.08
		*MMP16*	-4.81
		*ADAMTS9*	-5.86
		*POSTN*	-7.17
		*ELN*	-14.49
**PI3K–Akt/Rap1/actin**	PI3K-Akt signaling,	*SCIN*	9.04
**signaling rewiring**	Rap1 signaling,	*VAV3*	4.46
	regulation of actin cytoskeleton, intracellular signal transduction	*PIK3R1*	3.14
		*ADCY4*	2.47
		*RAC2*	2.38
		*FGF7*	2.33
		*ADCY3*	2.28
		*DIAPH3*	-3.31
		*IGF1*	-3.58
		*EGF*	-5.52
		*IQGAP3*	-8.53
**TGF-β superfamily rebalancing**	TGF-beta signaling pathway	*BMP6*	9.54
		*NOG*	6.04
		*DCN*	4.32
		*BMP2*	2.77
		*GDF5*	2.52
		*GDF6*	-3.28
		*INHBA*	-3.84
		*CDKN2B*	-4.49
		*INHBB*	-7.82
		*INHBE*	-19.17

A second module reflected extensive remodeling of extracellular matrix engagement and adhesion, supported by KEGG terms including ECM-receptor interaction, focal adhesion, cell adhesion molecules, and regulation of actin cytoskeleton, together with GO terms related to extracellular matrix organization, cell adhesion, external encapsulating structure, extracellular space, and integrin binding. Structural ECM and adhesion genes were broadly reduced in T1F2, including *COL1A1* (FC -4.29), *COL1A2* (-2.98), *FN1* (-2.87), *COMP* (-9.85), *THBS1* (-3.18), and *ITGA11* (-4.89). In parallel, the integrin profile shifted, with increased *ITGA8* (+6.86), *ITGB8* (+6.13), and *ITGA10* (+2.58), while *ITGA5* (-2.02) and *ITGA11* (-4.89) decreased, suggesting altered matrix engagement rather than simple loss of adhesion. This was accompanied by selective induction of matrix-remodeling and extracellular factors, including *MMP13* (+8.90), *MMP10* (+9.17), *CTSS* (+4.27), *C3* (+41.48), *CHI3L1* (+36.46), and *A2M* (+31.68). Collectively, these data indicate that T1F2 cells move away from a T1-associated fibro-adhesive matrix program toward a remodeled extracellular environment with altered integrin engagement and secreted factor composition.

The third module involved selective rewiring of intracellular signaling pathways. GO intracellular signal transduction and KEGG PI3K-Akt, Rap1, and TGF-β signaling were enriched, but showed mixed directional changes rather than uniform activation or suppression. A recurrent signaling hub was *PIK3R1* (FC +3.14), accompanied by increased *VAV3* (+4.46), *RAC2* (+2.38), *FGF7* (+2.33), *ADCY3* (+2.28), *ADCY4* (+2.47), and *SCIN* (+9.04), alongside decreased *IQGAP3* (-8.53), *DIAPH3* (-3.31), *EGF* (-5.52), and *IGF1* (-3.58). Within the TGF-β superfamily pathway, T1F2 cells showed increased *BMP2* (+2.77), *BMP6* (+9.54), *GDF5* (+2.52), *DCN* (+4.32), and *NOG* (+6.04) together with decreased *INHBA* (-3.84), *INHBB* (-7.82), *INHBE* (-19.17), *GDF6* (-3.28), and *CDKN2B* (-4.49), consistent with rebalancing away from an inhibin-associated T1 state toward a BMP/decorin/noggin-associated signaling state.

**Figure 5.**
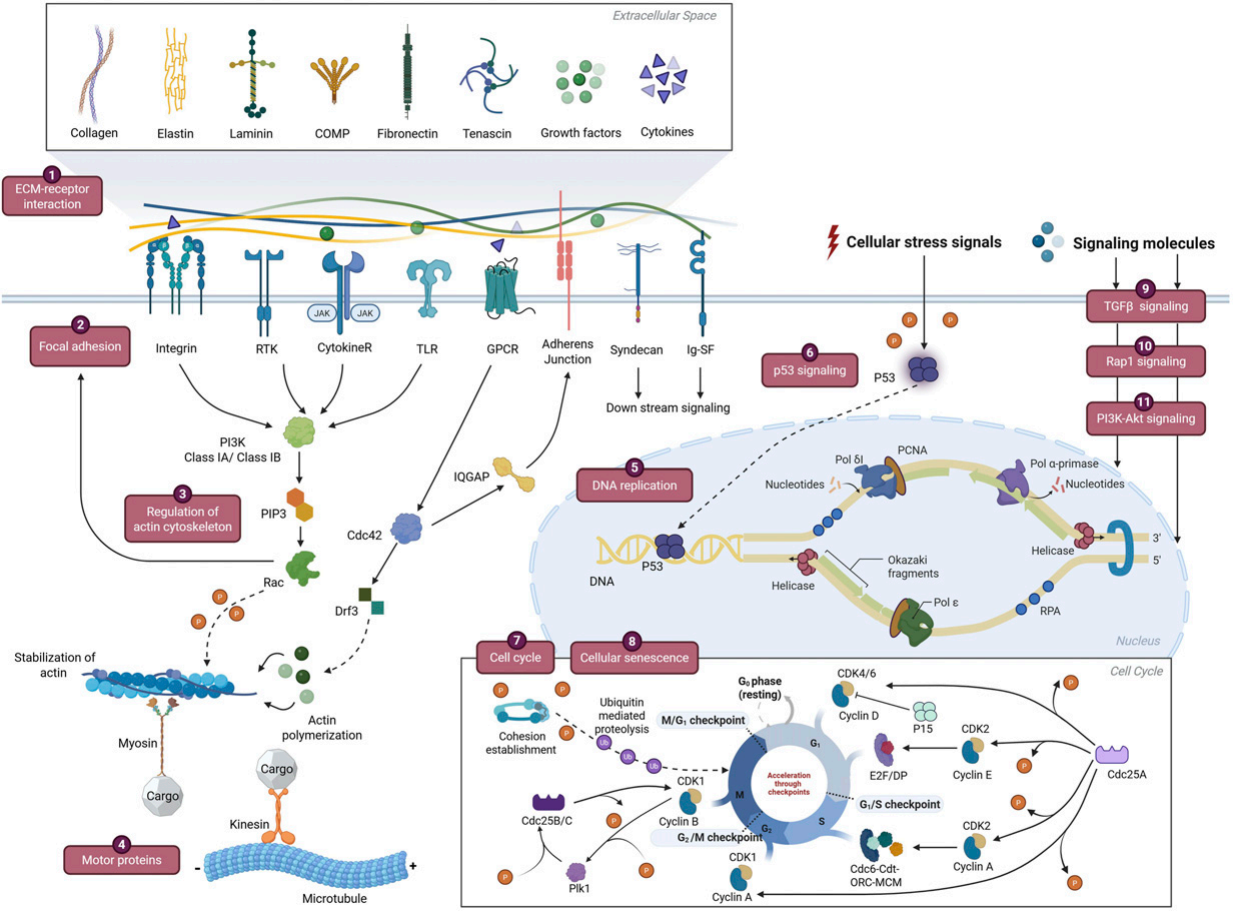
KEGG network model summarizing pathway remodeling induced by addition of FGF-2 to TGF-β1. Schematic network integrating the major KEGG pathways enriched in the TGF-β1 (T1) + FGF-2 (F2) priming versus T1 only RNA-seq comparison. The model summarizes an outside-in organization in which changes in extracellular ligands and matrix composition are linked to altered receptor and integrin engagement, selective rewiring of intracellular signaling, and downstream attenuation of DNA replication and cell-cycle machinery. Numbered modules correspond to (1) ECM-receptor interaction, (2) focal adhesion, (3) regulation of actin cytoskeleton, (4) motor proteins, (5) DNA replication, (6) p53 signaling, (7) cell cycle, (8) cellular senescence, (9) TGF-β signaling, (10) Rap1 signaling, and (11) PI3K-Akt signaling. The diagram is an integrative summary of the RNA-seq enrichment results; representative genes and fold changes are provided in Table 1 and Supplementary Table 2.

The KEGG network illustration summarizes these results as an outside-in pathway shift (Figure 5), in which extracellular matrix and ligand composition are remodeled at the tissue interface, the receptor/integrin layer is reconfigured, downstream PI3K-Akt/Rap1/TGF-β and actin-associated signaling is selectively rewired, and these changes converge on repression of DNA replication and cell-cycle machinery in the nucleus.

## Discussion

In this study, we provide an extensive characterization of the individual and combined effects of TGF-β1 and FGF-2 exposure during NC expansion, including how 2D monolayer transcriptional profiles relate to qualities of 3D tissue constructs formed thereafter. TGF-β1 is generally regarded for its ability to promote a cartilage-forming phenotype during expansion, while FGF-2 is used to promote division while still preventing dedifferentiation.^21,24,29^ Both attributes are essential to generating sufficient volumes of high-quality autologous cartilage grafts. Despite both factors being used extensively in cartilage tissue engineering, the molecular intricacies of their isolated and interactive effects have not yet been investigated to this extent.

Our results demonstrate that dual-priming exerted the greatest stimulatory effect on cumulative population doubling, followed by F2, with T1-treated cells showing the slowest proliferative rate. With the exception of Guerne et al., who reported TGF- β1 as a more potent mitogen,^30^ our data agree with previous authors’ comparisons of growth factors on human nasal and articular chondrocyte proliferation.^2,14,25,31^ At the time of molecular analysis, RNA sequencing revealed downregulation of genes involved in DNA replication and mitosis (*MCM4/5, CDK1, CCNB1/2, PLK1*) in T1F2-treated monolayer cultures relative to T1. In addition to the varying temporal profiles of growth factor effects,^32^ this illustrates the inherently sequential nature of cartilage formation, in which T1F2-treated cells were accelerated through the proliferative stage that occurs before condensation and matrix production.^33,34^ Although expansion often occurs at the cost of dedifferentiation into a more spindle-shaped fibroblastic phenotype,^13,14^ our results support existing literature that T1F2-treated cells retain the ability to redifferentiate in 3D culture systems, effectively producing tissue constructs that remain hyaline cartilage-like.^14,20,35,36^ Further, qualitative assessment of collagen II deposition appears more uniform in dual-primed pellets, aligning with earlier reports that the combination of T1 and F2 supports the production of cartilage-like tissue.^13,14,25^ Interestingly, however, dual-priming did not result in cartilage tissues that were broadly superior; we did not observe significantly different GAG/DNA ratios or mechanical properties,^25^ and the tissues formed from T1-treated cells exhibited more uniform Safranin-O staining, whereas T1F2-treated pellets were stained more intensely yet inconsistently.

In articular chondrocytes, FGF-2 has been shown to influence *MMP13* expression by activating multiple mitogen-associated (MAP) kinases appearing in the KEGG categories of PI3K/Akt and Rap1 signalling we found to be significantly enriched.^37,38^ Specifically, FGF-2 phosphorylation of FGFR1 activates p38 and JNK via protein kinase C delta (PKCδ), and ERK through Ras/Raf-mediated transduction, resulting in elevated *MMP13* expression.^17,38,39^ The role of FGF2 in cartilage metabolism is therefore nuanced, because while chronically elevated *MMP13* activity is implicated in pathological cartilage degradation typical of osteoarthritis, it is constitutively expressed in healthy cartilage and contributes to ECM turnover.^40–42^ FGF-2 can also activate the PI3K/Akt axis pathway to promote cell proliferation, survival, and control cartilage homeostasis through mammalian target of rapamycin (mTOR).^43–45^ Thus, we provide a schematic illustration of differentially regulated mechanisms to communicate how growth factor treatment not only influences basic biological aspects of cell cycle and DNA replication, but also ECM-receptor interactions influencing chondrogenesis.^46^ Together, without the associated finding of reduced GAG content in 3D pellets, the cumulative effect of pro-catabolic genes (*MMP10/13, CTSS*, *C3*, *CHI3L1*) expressed in T1F2-treated monolayers seems to be attenuated by protease inhibitors (*A2M* and *GDF5*) and mTOR signalling to support active ECM remodeling associated with adaptation to a 2D culture environment, rather than pathological degradation.^38,39,47^

## Effects of T1 priming on culture system adaptation

By interrogating the correlation between monolayer transcriptional profiles and those of 3D tissue constructs, we demonstrate that transcriptional profiles influenced by growth factor exposure are context-dependent. The relatively weak correlations between monolayer and pellet transcriptomic signatures likely reflect the fundamentally distinct culture environments that engage different cell interactions. Significant enrichment of ECM-receptor and focal adhesion modules underlies treatment-dependent responses as cells transition from a stiff, adhesion-driven environment to a 3D setting that introduces complex cell-matrix interactions and reduces cytoskeletal tension. From growth factor treatment in a 2D culture system, the widespread modulation of integrins (*ITGA5/A8/A10/B8)* and matricellular proteins (*TNC, THBS1, COMP)* primes differential mechanosensitivity to this environmental adjustment.^48–50^ In T1-treated monolayers, we observed concurrent downregulation of Rac/PI3K-associated genes (*RAC2, VAV3, PIK3R1*), which indicates a shift toward Rho signalling, as the two pathways are mutually antagonistic.^51^ Coinciding with upregulated contractile markers (*ACTA2, TAGLN*), this points to cytoskeletal dynamics promoting a more myofibroblast-like state in response to the stiff plastic substrate.^50,52^ Because *ACTA2* and *TAGLN* upregulation later resolve in 3D, this suggests that T1-priming heightens responsivity to environmental stiffness through modulated integrin-mediated signalling and mechanotransduction, consistent with prior *in vitro* studies and the self-reinforcing signalling of TGF-β1 in fibrotic diseases.^53–57^ Supporting this, Yan et al. recently demonstrated that TGF-β1 treatment potentiates stiffness-dependent responses in multipotent vascular stem cells—enhancing contractile marker expression on stiff substrates (20 kPa), and promoting chondrogenic markers on soft gels (1 kPa).^58^ Altogether, while prior 2D priming may still influence matrix deposition, as evidenced by sustained expression of fibrous ECM components such as *COL1* and *FN1*, our findings support that growth factor priming shapes, but does not fully determine, subsequent chondrogenic outcomes in 3D culture as the emergence of cell–cell and cell–ECM interactions introduces more self-regulated mechanisms of signalling.^59^ This aligns with previous findings that monolayer gene patterns only partly predict 3D construct profiles,^20,60^ and that high cell density 3D culture systems may be more influential than growth factors for maintaining or re-differentiating the chondrocyte phenotype.^20,61^

## Interactions between TGF-β1 and FGF-2 pathways

Finally, we demonstrate that FGF-2 treatment of NCs dominates the 2D molecular profile induced by combined growth factor supplementation with TGF-β1, whereby RNA sequencing reveals a greater similarity between T1F2 and F2 transcriptomes compared to T1F2 and T1. Both comparisons revealed modulation of genes classified under the ECM remodeling and intracellular signaling GO categories, although the enrichment scores were much lower for T1F2 than for F2 (Supplementary Figure 1). While minor, the synchronized enrichment of genes involved in ECM architecture could contribute to the increased tissue volume and qualitatively weaker Saf-O staining observed in F2-primed constructs. Overall, overlap of T1F2 and F2 groups along the primary sources of variance (PC1-PC2, 50.22%) indicates a more similar identity than with T1-treated cells, which is corroborated by the numbers of DEGs and heat map clustering. This complements the findings of Shi et al., that FGF-2 effects are prioritized over those of TGF-β1 when the two factors are used concurrently, whereby FGF-2 inhibits the action of TGF-β1 on IGF-I gene expression, and TGF-β1 synergistically stimulates FGF-2 expression.^32^ Dependent on the proteins recruited to the receptor-ligand complex, non-canonical TGF- β1 signalling can activate the same MAP kinases as FGFR1 phosphorylation by FGF-2 (ERK, p38, JNK), and the PI3K/Akt axis, likely mediating the interaction between both factors.^62^ Further, FGF-2 activation of ERK MAP kinases has also been shown to block nuclear localization of Smad2/3 via Ras to mitigate TGF-β1 signalling.^63,64^ To this end, we observe T1/F2 cross-talk promoting a balanced state of cartilage maturation through modulation of the bone morphogenetic protein (BMP) subfamily of the TGF-β signalling superfamily, which is important for regulating chondrocyte phenotype and stimulating cartilage matrix components *in vitro*.^65,66^ While previous authors used T1, F2, GDF5, and BMPs 2 and 6 to successfully drive stable cartilage formation and repair osteochondral defects in mice, we observed enhanced *BMP2/6* and *GDF5* expression from T1F2 priming alone.^47^ Consistent with their results, we observe enhanced *NOG* expression among T1F2-treated cells, supporting that Noggin signalling endogenously moderates BMP-induced chondrogenesis to regulate cartilage development.^47^ Considering the emergence of a similar molecular profile, our study suggests that T1 and F2 priming alone may be sufficient to achieve the same quality of cartilage.

## Limitations

Our investigation of growth factor interactions with cartilage biology and tissue engineering, while extensive, is not without its limitations. The choice not to include a control group was due to low cell yield and dedifferentiation in the absence of growth factors, preventing adequate pellet formation.^24,29^ It is for these same reasons that combined T1F2 treatment is considered common practice for NC tissue engineering and has been used in human clinical trials.^6–8,67^ Therefore, our experimental design was intended to evaluate the individual and interactive contributions of each factor relative to this standard condition. However, this precludes assessment of baseline chondrocyte signalling, restricting conclusions to comparative effects between growth factor conditions rather than absolute effects on chondrogenesis. Finally, the reliance on transcriptomic analyses without corresponding protein-level validation limits our insight into functional activity, as mRNA expression does not always correlate with protein abundance.^68–70^ Even so, the coordinated regulation of multiple genes across key signalling pathways, together with our tissue-level analyses of associated phenotypic changes, supports the biological relevance of these findings.

In the context of practical clinical considerations for nasal tissue engineering, the combination of TGF-β1 and FGF-2 has been used in clinical trials for nasal reconstruction and for repairing articular cartilage defects in the knee joint.^6–8^ While engineered constructs with higher sulfated GAG content, collagen II content, and elastic moduli are associated with superior clinical outcomes,^8^ these trials all note a high degree of inter-patient variability in these measures, which is reflected in our T1F2 constructs.^7,8^ However, we provide a novel and in-depth characterization of how these factors interact from the molecular to tissue levels to support efforts to address this clinical problem. From dual priming, we describe a pro-chondrogenic transcriptional program supported by enhanced BMP signalling balanced by concurrent Noggin signalling, which supports the use of T1F2 for generating phenotypically stable constructs for transplantation even if the cartilage quality is not significantly superior by all metrics. Our results suggest that while the greater volume and proliferation rate of F2-primed pellets may be advantageous in lowering the culture time required for repairing larger defects, priming with T1 alone improves consistency of mechanical properties and Safranin-O staining, which could support more reproducible results but may also be more sensitive to the mechanical context of the culture environment through modulated integrin expression. This study provides foundational insight into the complex and multifaceted effects of growth factor treatment on chondrocyte biology, highlighting treatment-dependent transcriptional regulation of cartilage synthesis and environmental adaptation. Emphasizing the complex influences of media additives and culture conditions on overall cell signalling, further research is needed to inform clinical practice guidelines to optimize and standardize manufacturing processes in tissue engineering.

## Supplemental material

Supplemental material - FGF-2 signaling dominance in TGF-β1/FGF-2–primed nasoseptal chondrocytes for cartilage tissue engineering Supplemental material for FGF-2 signaling dominance in TGF-β1/FGF-2–primed nasoseptal chondrocytes for cartilage tissue engineering by Zhiyao Ma, Kiarra Grimes, Aillette Mulet-Sierra, Melanie Kunze, Kezhou Wu, Adetola B. Adesida in Journal of Tissue Engineering

Supplemental material - FGF-2 signaling dominance in TGF-β1/FGF-2–primed nasoseptal chondrocytes for cartilage tissue engineering Supplemental material for FGF-2 signaling dominance in TGF-β1/FGF-2–primed nasoseptal chondrocytes for cartilage tissue engineering by Zhiyao Ma, Kiarra Grimes, Aillette Mulet-Sierra, Melanie Kunze, Kezhou Wu, Adetola B. Adesida in Journal of Tissue Engineering

Supplemental material - FGF-2 signaling dominance in TGF-β1/FGF-2–primed nasoseptal chondrocytes for cartilage tissue engineering Supplemental material for FGF-2 signaling dominance in TGF-β1/FGF-2–primed nasoseptal chondrocytes for cartilage tissue engineering by Zhiyao Ma, Kiarra Grimes, Aillette Mulet-Sierra, Melanie Kunze, Kezhou Wu, Adetola B. Adesida in Journal of Tissue Engineering

## Data Availability

The datasets presented in this study are available in the GEO online repositories under accession number: GSE329525.*
