# Supplementary material for: FGF-2 signaling dominance in TGF-β1/FGF-2–primed nasoseptal chondrocytes for cartilage tissue engineering
Source: J Tissue Eng. 2026 Aug 13;17:20417314261479435. doi: 10.1177/20417314261479435 (PMC13473784; doi:10.1177/20417314261479435)
Supplement: Supplementary file 1 [file sj-pdf-1-tej-10.1177_20417314261479435.pdf]

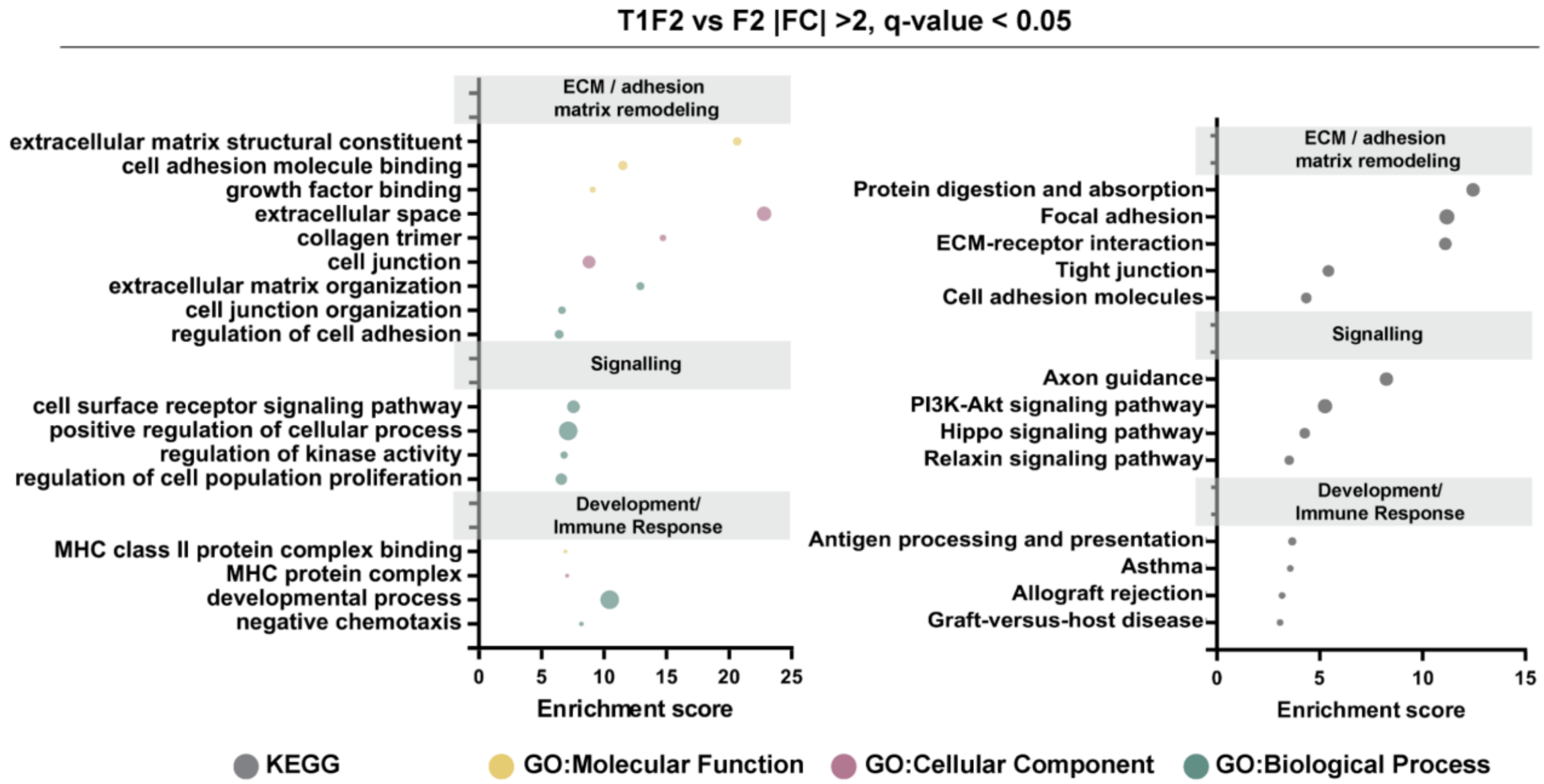

Supplementary Figure 1. Selected non-redundant GO terms and enriched KEGG pathways for the T1F2 vs F2 comparison, grouped into three functional modules: ECM/adhesion matrix remodeling, signalling, and development/immune response. KEGG terms are shown in grey; GO terms are color-coded by ontology.
