# Supplementary material for: FGF-2 signaling dominance in TGF-β1/FGF-2–primed nasoseptal chondrocytes for cartilage tissue engineering
Source: J Tissue Eng. 2026 Aug 13;17:20417314261479435. doi: 10.1177/20417314261479435 (PMC13473784; doi:10.1177/20417314261479435)
Supplement: Supplementary file 2 [file sj-pdf-2-tej-10.1177_20417314261479435.pdf]

Supplementary Table 1. Detailed primer sequence information of genes analyzed by RT-qPCR

Gene		Primer Sequences	Accession number
RPL13A	Forward	CCTGGAGGAGAAGAGGAAAGAGA	NM_012423.4
	Reverse	TTGAGGACCTCTGTGTATTTGTCAA	
B2M	Forward	TGCTGTCTCCATGTTTGATGTATCT	NM_004048.2
	Reverse	TCTCTGCTCCCCACCTCTAAGT	
YWHAZ	Forward	TCTGTCTTGTCACCAACCATTCTT	NM_003406
	Reverse	TCATGCGGCCTTTTTCCA	
ACAN	Forward	AGGGCGAGTGGAATGATGTT	NM_001135.3
	Reverse	GGTGGCTGTGCCCTTTTTAC	
COL1A2	Forward	GCTACCCAACTTGCCTTCATG	NM_000089.3
	Reverse	GCAGTGGTAGGTGATGTTCTGAGA	
COL2A1	Forward	CTGCAAAATAAAATCTCGGTGTTCT	NM_033150
	Reverse	GGGCATTTGACTCACACCAGT	
COL10A1	Forward	GAAGTTATAATTTACACTGAGGGTTTCAAA	NM_000493.3
	Reverse	GAGGCACAGCTTAAAAGTTTTAAACA	
SOX5	Forward	ATCCCAACTACCATGGCAGCT	NM_006940.6
	Reverse	TGCAGTTGGAGTGGGCCTA	
SOX6	Forward	GCAGTGATCAACATGTGGCCT	NM_017508.3
	Reverse	CGCTGTCCCAGTCAGCATCT	
SOX9	Forward	CTTTGGTTTGTGTTCGTGTTTTG	NM_000346.3
	Reverse	AGAGAAAGAAAAAGGGAAAGGTAAGTTT	
ACTA2	Forward	TGCCTGATGGGCAAGTGA	NM_001141945.2
	Reverse	CTGGGCAGCGGAAACG	
TAGLN	Forward	GGCATGAGCCGCGAAGT	NM_001001522.2
	Reverse	TCCTCCAGCTCCTCGTCATACT	
